# Microbial community and performance of a partial nitritation/anammox sequencing batch reactor treating textile wastewater

**DOI:** 10.1016/j.heliyon.2021.e08445

**Published:** 2021-11-20

**Authors:** Elisa Clagnan, Lorenzo Brusetti, Silvia Pioli, Simone Visigalli, Andrea Turolla, Mingsheng Jia, Martina Bargna, Elena Ficara, Giovanni Bergna, Roberto Canziani, Micol Bellucci

**Affiliations:** a Free University of Bolzano, Faculty of Science and Technology, Piazza Università 1, 39100 Bolzano, Italy; b Politecnico di Milano, Department of Civil and Environmental Engineering (DICA), Piazza L. da Vinci 32, 20133 Milano, Italy; c Lariana Depur Spa, Via Laghetto 1, 22073 Fino Mornasco, Italy

**Keywords:** Partial nitritation/anammox, Textile wastewater, Next-generation sequencing, Quantitative polymerase chain reaction (qPCR), Nitrogen removal

## Abstract

Implementation of onsite bioremediation technologies is essential for textile industries due to rising concerns in terms of water resources and quality. Partial nitritation-anaerobic ammonium oxidation (PN/A) processes emerged as a valid, but unexplored, solution. In this study, the performance of a PN/A pilot-scale (9 m^3^) sequencing batch reactor treating digital textile printing wastewater (10–40 m^3^ d^−1^) was monitored by computing nitrogen (N) removal rate and efficiencies. Moreover, the structure of the bacterial community was assessed by next generation sequencing and quantitative polymerase chain reaction (qPCR) analyses of several genes, which are involved in the N cycle. Although anaerobic ammonium oxidation activity was inhibited and denitrification occurred, N removal rate increased from 16 to 61 mg N g VSS^−1^ d^−1^ reaching satisfactory removal efficiency (up to 70%). Ammonium (18–70 mg L^−1^) and nitrite (16–82 mg L^−1^) were detected in the effluent demonstrating an unbalance between the aerobic and anaerobic ammonia oxidation activity, while constant organic N was attributed to recalcitrant azo dyes. Ratio between nitrification and anammox genes remained stable reflecting a constant ammonia oxidation activity. A prevalence of ammonium oxidizing bacteria and denitrifiers suggested the presence of alternative pathways. PN/A resulted a promising cost-effective alternative for textile wastewater N treatment as shown by the technical-economic assessment. However, operational conditions and design need further tailoring to promote the activity of the anammox bacteria.

## Highlights

• The feasibility of the PN/A process to treat N-rich textile wastewater was evaluated.
• A pilot scale SBR (V = 9 m3) was installed in a DTP company and monitored for 8 weeks.
• The NRR reached 70 mg N_TOT_ g VSS^−1^ d^−1^ and an efficiency of 70%.
• NGS data suggest that nitrification-denitrification was established during start-up.
• An onsite PN/A SBR plant could lead to a 40% saving on wastewater disposal cost.

## 1 Introduction

Textile industries are a major concern for water quality. They consume large quantities of water to process fabric (∼200 L per kg of fabric produced [1, 2]) while generating large amount of wastewater (17–20% of total industrial wastewater [2]). The introduction of digital textile printing (DTP) technologies decreases the environmental impact of the textile district whilst providing high quality standards in a shorter production time. Nevertheless, ink-jet printing techniques require the pre-soaking of fabrics in urea to fix dyes, with the drawback of releasing wastewater with higher (+200%) concentration of N than that of effluents from conventional printing technologies. Additionally, extremely toxic compounds, such as dyes, textile auxiliaries and heavy metals are also found in this type of wastewater. Extensive treatments targeting the specific compounds, including advanced physical, chemical, biological and/or combination of them, are therefore necessary prior to discharge to reduce damage to environment and human health. New techniques for wastewater purification are constantly being improved [3, 4, 5]. Microbial treatment technologies are considered as the “next-generation ecological ditches” as they are considered effective and economical for the simultaneous removal of N, phosphorus (P) and heavy metals from wastewaters such as the textile [6, 7, 8, 9]. Conventional biological N removal process for such N-rich wastewater requires intensive aeration and organic inputs/substrates, with the disadvantage of high consumption of energy, production of large amounts of sludge and massive emission of greenhouse gases. Recovery efficiencies and energy requirements of different bioremediation techniques are however often disregarded and there is a need for the consideration of the impact of these techniques when applied at full-scale [10]. The development of alternative cost-effective bioremediation technologies is therefore essential to promote an environmentally sustainable textile industry.

Completely autotrophic N removal processes (ANR) emerged as an efficient and cost-effective alternative to conventional N removal processes for ammonia-rich wastewater, including textile wastewater from DTP companies [11, 12, 13]. ANR combines the partial nitritation (PN) and anammox processes through the activity of ammonium oxidizing bacteria (AOB) and anammox bacteria (AMX). The two guilds of microbes are commonly aggregated in the form of granule or biofilm with an external layer composed mainly of AOB and a core of AMX [14, 15, 16, 17]. Within the granule, aerobic AOB oxidise ammonium (NH_4_^+^) to nitrite (NO_2_^-^) through the reaction of nitritation while creating an anoxic condition by consuming oxygen. Low dissolved oxygen concentration (DO) combined with the accumulation of NO_2_^-^ promotes the growth of AMX, which convert the remaining NH_4_^+^ and NO_2_^-^ into nitrate (NO_3_^-^) and dinitrogen gas (N_2_) [18,19]. In the PN/A process, the oxygen demand is lower and external carbon source are not needed. Nevertheless, the main difficulty for the application of a PN/A process is the long start up time required because of the slow growing AMX and their low versatility to changes of operational and environmental conditions [20, 21, 22].

To develop an effective and resilient ANR process, the factors affecting the community structure of the PN/A granules need to be understood [23]. The main drivers found to shape the community are temperature, salt concentration and level of chemical and biochemical oxygen demand (COD and BOD) [22, 24]. Communities have proven to be stable under constant conditions; however, they easily shift according to variations of operational parameters, environmental conditions, and the composition of the wastewater during running periods [25, 26]. Therefore, composition, abundance and evolution of AOB and AMX along with their competitors (such as nitrite oxidizing bacteria (NOB), which carry out the oxidation of NO_2_^-^ to NO_3_^-^, and denitrifiers), should be better correlated with the characteristic of the textile wastewater and the operational parameters of the technology. The selection of an efficient community and parameters within a sequential bioreactor has additionally the potential to minimize N_2_O production in such wastewater treatment plants [27].

To date, limited research has been done on microbial communities of textile wastewater treatment plants [24, 26]. To characterise the microbial community of the PN/A process, various genes encoding for enzymes of the N-cycle have been often used. To identify and quantify AOB and AMX through Real-Time Polymerase Chain Reaction (qPCR) based methods, the functional genes coding for ammonia monooxygenase (*amoA*) and hydrazine oxidoreductase (*hzo*), respectively, are commonly used [14, 28]. Whereas the two variants of the enzyme for nitrite reductase, *nirK* and *nirS*, are often used for the detection of denitrifiers [29, 30], together with the gene *nosZ* (for the enzyme nitric oxide synthetase), recently found in two clades [31, 32, 33]. Improved primers are constantly being introduced; however, no primer combination can amplify all known sequences while maintaining an acceptable level of specificity [34]. The use of next generation sequencing technology could provide more information on the structure, dynamics and interaction of and between original wastewater and granulated sludge microbial communities, highlighting the dominant microorganisms (e.g. *Planctomycetes* and *Thaumarchaeota*) responsible for the textile wastewater purification process [24].

To better understand microbial dynamics and improve the understanding on the efficiency of the ANR process for the bioremediation of textile wastewater, the performance of a pilot-scale sequencing batch reactor (SBR), treating real DPT wastewater through a PN/A process, was evaluated for 8 weeks. The N removal rate and efficiency of the SBR were then correlated to the community structure (abundance and composition of the main populations) and to the abundance of N cycle related genes within the granular biomass to gain insights into the biochemical pathways occurring in the reactor. A techno-economic analyses was also performed to calculate the potential saving of the onsite proposed process and for scale-up perspective.

## 2 Material and methods

### 2.1 Pilot plant installation and operation

The pilot plant was located in a textile printing company in the province of Como (Northern Italy) for the treatment of industrial textile wastewater through the PN/A process. The company operates 28 ink-jet printers (printing capacity of 9,000 000 m year^−1^) and discharges more than 380 000 m^3^ y^−1^ of wastewater.

**Figure 1:**
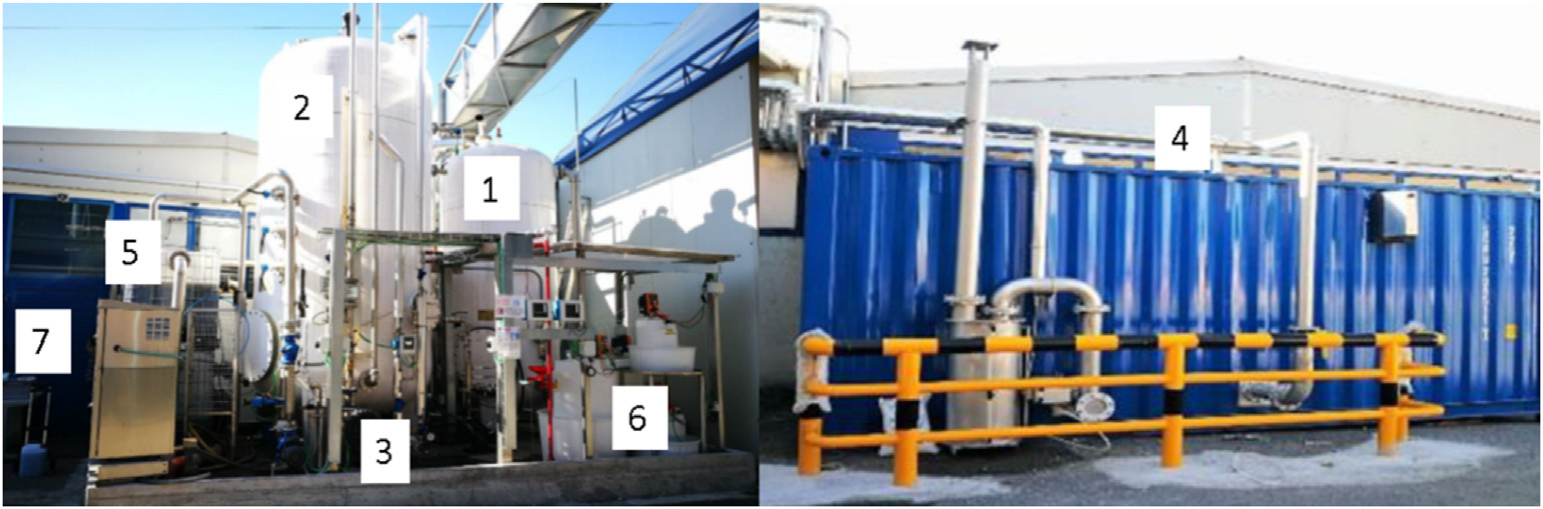
Pilot plant. 1 - feeding tank for untreated wastewater, 2 - PN/A reactor, 3 - pH, DO, ORP, ammonia and nitrate probes, 4 - gasometer, 5 - in-series tanks accumulating the discharge of the SBR, 6 - storage and dosing system, 7 - PLC. More details in the main text. Photos by Martina Bargna, at Stamperia di Cassina Rizzardi (SCR).

The pilot plant was designed in order to have a potential treatment capacity of 40 m^3^ y^−1^ and a scheme is reported in Figure 1 and includes: 1) a feeding tank (V = 6 m^3^) for untreated wastewater equipped with a basket filter at the entrance to prevent the entry of any threads. A dedicated recirculation pump keeps the stored wastewater mixed, while the temperature is kept at 35°-38 °C thanks to a cooling/heating system; 2) a PN/A SBR reactor composed of a tank (volume = 12 m^3^; working volume = 9 m^3^), feeding and discharge pumps, and a gas recirculation system for sludge mixing. Pumps for feeding, discharge and recirculation of the sludge and the gas recirculation blower were equipped with an inverter to allow for flow rate variations. The tank was also equipped with a valve for manual sludge extraction and reagent dosing, as well as an heating system for keeping the temperature constant; 3) probes for online monitoring of pH, dissolved oxygen (DO), RedOx potential (ORP), NH_4_^+^ and NO_3_^-^, and temperature; 4) a gasometer for the volumetric compensation of the gas phase. This was also equipped with a hydraulic guard to maintain the pressure inside the SBR at around 30–50 relative millibars; 5) two tanks in-series for storing the SBR discharge; 6) a storage and dosing system for chemical reagents (i.e. acid, base, phosphorus and antifoam); 7) an automation and remote control system (PLC), which regulates reactor operations on the basis of feedback information given by the probes.

The reactor was inoculated with PN/A granular biomass (Paques, the Netherlands) (Total Suspended Solids (TSS) = 1.73 g L^−1^, Volatile Suspended Solids (VSS) = 1.34 g L^−1^, VSS/TSS = 77.4%). The SBR cycle was composed of four phases: 1) feeding, the wastewater enters the system and gets in contact with the granular biomass (45 min); 2) reaction, the wastewater and the granules are mixed to facilitate biochemical reactions (311 min); 3) sedimentation of bacterial granules (12 min); 4) discharge of the treated effluent and stand-by (8 min). The number of cycles for day was set equal to 4 (6 h each). The reactor has been monitored for 8 weeks; during the first five weeks, 1000 L of feeding wastewater has been exchanged with the reactor suspension every cycle, while for the last three weeks, the volume exchanged per cycle was 1300 L. Averages of physiochemical parameters of the feeding wastewater are reported in Table 1. The pH was kept in the 7.3–8.4 range, while compressed air was sparged to keep DO in the range of 0.5–0.7 mg L^−1^ and the NH_4_^+^ content within 20–50 mg L^−1^.

**Table 1:** Comparison of the chemical data between the feeding wastewaters and the SBR effluent across the eight experimental weeks. Data represent average values (±standard deviation).

Time	Feeding wastewater [mg/L]							SBR effluent [mg/L]							pH		Conductivity [μS/cm]	
	NO_2_^-^-N	NO_3_^-^-N	NH_4_^+^-N	COD	N_org_-N	P_tot_	TSS	NO_2_^-^-N	NO_3_^-^-N	NH_4_^+^-N	COD	N_org_-N	P_tot_	TSS	In	Out	In	Out
Week 1	0 (±0)	0 (±0)	162.0 (±13.1)	756.0 (±0.0)	35.0 (±0.0)	3.0 (±0.0)	101.0 (±0.0)	43.6 (±18.9)	1.3 (±0.8)	72.0 (±32.4)	546.0 (±0.0)	26.3 (±0.0)	2.6 (±0.0)	92.0 (±0.0)	9.1 (±0.0)	7.9 (±0.2)	2093.7 (±26.6)	2433.3 (±75.9)
Week 2	0 (±0)	0 (±0)	163.0 (±12.0)	647.0 (±0.0)	35.0 (±0.0)	2.6 (±0.0)	106.0 (±0.0)	82.0 (±8.9)	3.4 (±0.0)	34.5 (±1.5)	507.0 (±0.0)	24.0 (±0.0)	2.4 (±0.0)	90.0 (±0.0)	9.1 (±0.1)	7.9 (±0.1)	2148.0 (±20.0)	2380.0 (±40.0)
Week 3	0 (±0)	0 (±0)	163.0 (±13.5)	666.0 (±37.0)	33.0 (±2.0)	2.5 (±0.3)	107.5 (±3.5)	54.5 (±10.7)	6.2 (±2.9)	35.3 (±2.6)	474.0 (±16.0)	27.0 (±4.0)	2.3 (±0.1)	74.5 (±6.5)	8.8 (±0.2)	7.7 (±0.0)	2265.0 (±75.0)	2415.0 (±35.0)
Week 4	0 (±0)	0 (±0)	165.7 (±15.5)	720.0 (±16.5)	36.6 (±6.6)	3.4 (±1.1)	253.0 (±0.0)	26.9 (±3.4)	2.2 (±1.4)	31.3 (±2.1)	456.5 (±27.5)	27.2 (±0.8)	2.3 (±0.2)	74.0 (±0.0)	8.8 (±0.0)	7.8 (±0.0)	2246.5 (±73.5)	2240.0 (±30.0)
Week 5	0 (±0)	0 (±0)	182.7 (±20.3)	729.5 (±14.5)	25.5 (±5.5)	3.1 (±0.2)	91.0.0 (±5.0)	31.8 (±7.4)	1.2 (±0.1)	35.8 (±3.3)	483.5 (±4.5)	25.5 (±3.5)	3.1 (±0.1)	82.0 (±15.0)	8.7 (±0.2)	7.7 (±0.1)	2282.5 (±274.6)	2492.0 (±117.1)
Week 6	0 (±0)	0 (±0)	196.5 (±0.5)					15.5 (±4.9)	0.9 (±0.2)	42.5 (±4.5)								
Week 7	0 (±0)	0 (±0)	200.3 (±15.2)	843.0 (±0.0)	19.7 (±2.7)	3.4 (±0.0)	49.0 (±0.0)	28.3 (±7.7)	1.3 (±0.1)	15.7 (±3.4)	490.0 (±0.0)	24.0 (±0.0)	2.9 (±0.0)	57.0 (±0.0)	8.8 (±0.1)	7.6 (±0.2)	2183.0 (±27.0)	2216.5 (±43.5)
Week 8	0 (±0)	0 (±0)	173.0 (±17.0)		25.0 (±0.0)			18.8 (±5.4)	1 (±0.1)	18.0 (±3.0)		20.0 (±0.0)			8.6 (±0.0)	7.3 (±0.1)	2076.5 (±91.5)	2137.0 (±93.0)

Probes within feeding tank and reactor detected and recorded DO, pH, ORP, NH_4_^+^-N, NO_3_^-^-N, pressure, temperature and volume every minute, as well as the number of cycles per day, the quantity of wastewater exchanged at each cycle, the volume of the basic and acid solutions dosed and the air sparged.

During feeding and discharge phases, samples of the wastewater at the inlet (in) and outlet (out) of the SBR were collected to carry out physicochemical and microbiological analyses.

### 2.2 Physicochemical analyses

Samples of the feeding wastewater and of the effluent of the reactor were characterised to assess and monitor the performance of the pilot plant. Two automatic samplers were installed to collect 350 mL of wastewater during the feeding and discharge phases of every cycle. The sampling bottles were changed at the beginning of each new cycle. The concentration of total N (N_TOT_), NH_4_^+^-N, NO_3_^-^-N, NO_2_^-^-N, Total Kjeldahl Nitrogen (TKN), COD, TSS and total phosphorus (P_TOT_) were measured in the inlet and outlet in duplicate according to APAT IRSA methods and Standard Methods [35]. Organic N (N_org_-N) was determined as the difference between the TKN and NH_4_^+^-N. The volatile suspended solids of the granular biomass in the reactor were measured according to the Standard Methods [35]. The conductivity and pH were measured with a conductometer (HQ440d, Hach-Lange) and a pH meter (IONCHECK10, Radiometer Analytical), respectively. The concentrations of Cu, Cr, Cd, Pb and Ni were determined by Graphite Furnace Atomic Absorption Spectrometry (GFAAS; SIMAA 6000, PerkinElmer) (IRSA-CNR: 3250, 3150,3220), while Zn, K, Mg, Ca, Si, Fe, Na, Mn, Mo and Al by Inductively Coupled Plasma-Optical Emission Spectroscope (ICP-OES; Optima 7000 DV PerkinElmer, Software control WinLab) (IRSA-CNR 4020 and 3030).

Total N loading rate (NLR), removal rate (NRR) (mg Ntot/gVSS/d) and nitrogen removal efficiency (η) were computed by Eqs. (1), (2), and (3).

[1] NLR = N_TOTin_ [mg L^−1^] · (V_exc_ [L cycle^−1^] · number of cycle per day [cycle d^−1^]) / (Biomass concentration [g VSS L^−1^] · V reactor [L])

[2] NRR = (N_TOTin_ [mg L^−1^] - N_TOTout_ [mg L^−1^]) · V_exc_ [L cycle^−1^] · number of cycle per day [cycle d^−1^] / (Biomass concentration [g VSS L^−1^] · V reactor [L])

[3] η = NRR/NLR

### 2.3 Microbial community analyses

#### 2.3.1 DNA extraction

Biomass (10 mL) was collected inside the reactor at the end of each week (n = 8) and stored at -20 °C prior to the molecular analyses. From each sample (500 μL–540 mg of wet and ∼0.05 mg of dry sludge), DNA was extracted in triplicate using the DNeasy PowerSoil kit (QIAGEN, Germany) according to manufacturer guidance except for the first step, which was executed by vortexing the samples in an Eppendorf ThermoMixer Comfort (Germany) at 1400 rpm for 10 min. The yield of the purified DNA was quantified using Qubit™ (Thermo Fisher Scientific, USA), while possible fragmentation was determined through gel electrophoresis 1% (w/v) 1×TAE agarose gels. DNA was then stored at -80 °C until further analyses. Prior to qPCR and 16S NGS, the DNA extracted from the triplicate were pulled together to reduce biases due to the extraction step.

#### 2.3.2 qPCR

Quantitative PCR amplifications were carried out in triplicate using the Rotor-Gene SYBR Green PCR Kit (QIAGEN, Netherlands) according to manufacturer's instruction. Extracts were diluted 1:5 in nuclease-free water to reduce possible inhibition. An aliquot of 3 μL of a 1:5 solution of template was added per reaction to the qPCR master mix. The conditions of the qPCR followed the protocols outlined in Table S1. Reactions were run on a Rotor Gene real-time PCR cycler (QIAGEN, Netherlands) according to manufacturer's instructions.

Triplicate curves were built using corresponding DNA standard template (from 10^8^ to 10^2^ copy numbers, 10-fold serial dilution series). Standard curves were produced for absolute quantifications of four bacterial denitrification genes (*nirS, nirK, nosZ1 and nosZ2*), one for bacterial and archaeal ammonium oxidizers (AOA) (*amoA*) and one for bacterial anammox (*hzo* cluster 1). DNA Standards were synthesised by GeneArt Gene Synthesis (Thermo Fisher Scientific, Germany). The synthetic standards were created, after an in-silico analyses, by inserting the synthetic gene target sequence followed by a restriction site for PvuII within a vector plasmid. Standard plasmid was linearized with the restriction enzyme PvuII (Thermo Fisher Scientific, Germany), purified through PureLink™ (Invitrogen – Life Technologies, Germany) and quantified though the use of Qubit® dsDNA HS Assay Kit (Molecular Probes– Life Technologies, USA) following manufacturer's instructions.

#### 2.3.3 NGS of the 16S rRNA gene and bioinformatic

Genomic DNA of each sample were sequenced at Stab Vida lda (Lisbon, Portugal). Sequencing was performed by targeting the V5 and V7 regions of the 16S rRNA gene using the primers 799F (5′-AACMGGATTAGATACCCKG-3′) [36] and 1175R1 (5′-ACGTCRTCCCCDCCTTCCT-3′) [37]. The generated DNA libraries were then sequenced with MiSeq Reagent Kit Nano on the lllumina MiSeq platform, using 250 bp paired-end sequencing reads. The nucleotide sequences generated and analysed are available at the NCBI SRA repository (BioProject accession number: PRJNA623880).

The sequences resulting from the NGS were quality checked through the FastQC software and analysed using DADA2 [38]. DADA2 was used as per https://benjjneb. github.io/dada2/tutorial.html. Reads were truncated at 230 (forward) and 200 (reverse) to remove the low-quality section of the reads. Additionally, the adapter sequence was removed with the trimLeft function set at 19 (length of the primers) for both forward and reverse reads. For taxonomic assignment, the SILVA database was used as reference.

Statistical analyses were performed with R (version 3.6.2). The final data matrix was normalized, and rarefaction curves were built. The overall community structure was analysed with detrended correspondence analysis (DCA) that showed a linear response (axis length >4 SD) [39, 40]. A principal component analysis (PCA) was used to infer time patterns in species composition. Richness, diversity and evenness were calculated per each sample. Differences in samples were analysed through one-way analysis of variance (ANOVA) (p < 0.05) followed by Tukeýs post hoc test. Spearman rho correlation was used to analyse the influence of environmental factors on the logarithmic of the gene numbers.

### 2.4 Cost calculation

A cost and saving assessment due to the implementation of the onsite pilot plant was performed. The disposal cost of the raw and the PN/Annamox treated wastewater to the centralized WWT plant was calculated, and compared, by considering the unified tariff of the water companies in the area of Como [41]. Such document defines the disposal cost for domestic and industrial wastewaters based on the chemical-physical characteristics and discharge load. Therefore, the potential annual saving of the company was computed by considering the COD and N removal efficiencies of the existing onsite SBR.

An estimation of the capital and annual operational costs, required to scale up the system, was also done. The capital investment (CAPEX) is the cost for construction, design and construction management. The total construction investment, including the civil and electro-mechanical works, was calculated either as a function of the wastewater volume to be processed on a (average) daily basis (Total investment [€] = 4147.5 (Volume treated [m^3^/d])^0.4479^) or as a function of the nitrogen load in wastewater to be processed on a (average) daily basis (Total investment [€] = 101145 (N load treated [kg N/d])^0.4528^). Details on the derivation of these equations are reported in the supplementary materials (Table S2 and Figure S1). The construction and operation of an SBR plant able to fully treat the wastewater originated by the textile company (380 000 m^3^/y, 203 kg N/d) was therefore hypothesized. The average value of the two methods was considered. A 6.5% on the initial investment plus the cost of 140 h of technician (50 €/h) was assumed for design costs as standard design was already available. A cost of 4.0 % of the construction investment plus the cost of 240 h of technicians (50 €/h) was assumed for construction management/supervision and safety.

The operational costs of the plant are related to the annual maintenance, reagents, energy and water consumptions, and management and start-up. A detailed list of each cost is reported in Table S3. All costs were inferred based on the geographical contexts (Italian textile districts) and market prices for labour, civil, electrical and mechanical works.

## 3 Results and discussion

### 3.1 Performance of the SBR

During the experimental period inlet wastewater was characterised by negligible NO_2_^-^-N (0–5 mg L^−1^) and NO_3_^-^-N while NH_4_^+^-N ranged between 118 and 221 mg L^−1^ and organic N between 10 and 78 0 mg L^−1^ for a total N of 125–240 mg L^−1^ (Table 1). COD in the inlet wastewater ranged from 500 to 843 mg L^−1^ while total P between 0.96 and 4.49. Suspended solids and conductivity were in the range of 49–253 mg L^−1^ and 1108–2730 μS respectively. Additional average values for other elements were K: 3.92 (±0.78) mg/L, Mg: 6.70 (±1.40) mg/L, Ca: 30.76 (±6.85) mg/L, Si: 3.15 (±3.12) mg/L, Fe: 0.09 (±0.04) mg/L, Na: 257.44 (±35.14) mg/L, Zn: 0.08 (±0.02) mg/L, Mn: 0.01 (±0.00) mg/L Mo: 0.00 (±0) mg/L, Al: 0.03 (±0.00) mg/L, Cr: 36.26 (±30.58) μg/L, Ni: 6.14 (±5.03) μg/L, Pb: 0.05 (±0.00) μg/L, Cu: 32.27 (±17.74) μg/L, Cd: 0.00 (±0) μg/L.

**Figure 2:**
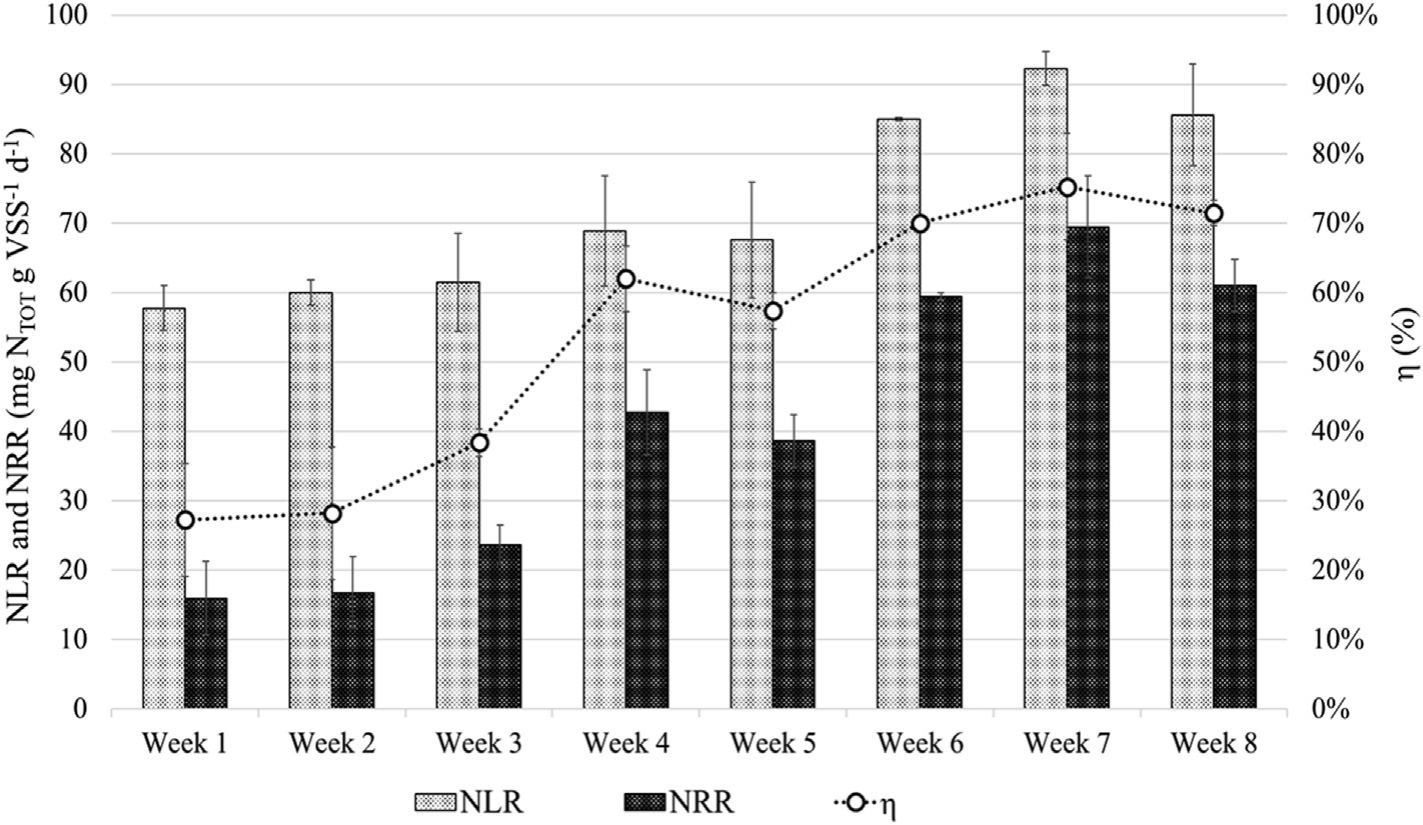
Trends of the N loading (NLR) and removal (NRR) rates, as well as the N removal efficiency (η), of the SBR.

**Figure 3:**
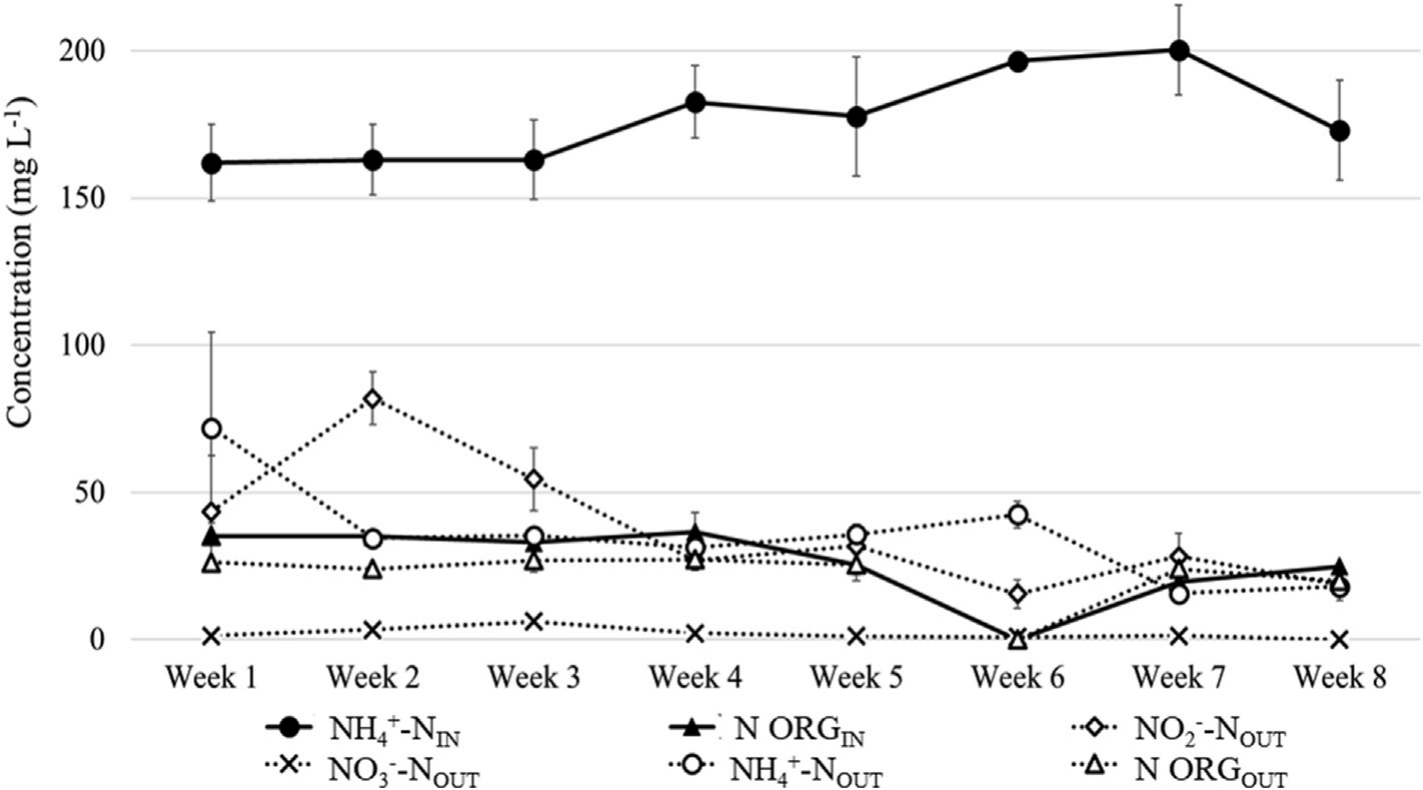
Concentration of the N compounds in the inlet and outlet over time.

The N removal rate (NRR) and efficiency (η) of the SBR were monitored over a period of 8 weeks. As shown in Figure 2, the NLR increased from 60 ± 2 mg N_TOT_ g VSS^−1^ d^−1^ (first three weeks) to 88 ± 4 mg N_TOT_ g VSS^−1^ d^−1^ (last three weeks), while the NRR increased up to 70 mg N_TOT_ g VSS^−1^ d^−1^ reaching a removal efficiency of 70% during the last three weeks. The findings are in agreement with those reported in lab scale studies applying the PN/A processes for the nitrogen removal in textile wastewater (η = up to 79%), in animal feed processing wastewater (η = up to 79%), and in diluted food waste digestate supplemented with nitrite (NLR = 98.8 N_TOT_ g m^−3^ d^−1^, NRR = 77.5 N_TOT_ g m^−3^ d^−1^, η = 76%) [12, 42, 43], and support the feasibility of the PN/A for the treatment of recalcitrant industrial wastewater. Nevertheless, Figure 3 shows that a residue of NH_4_^+^-N (between 18 and 70 mg L^−1^) and NO_2_^-^-N (between 16-82 mg L^−1^) were detected in the effluent, indicating that the ink-jet textile wastewater contained some undetected toxic compounds, which inhibited the anammox bacterial activity as previously reported by [44]. The NO_3_^-^-N level in the effluent was negligible, while the COD ranged between 629 and 843 mg L^−1^ in the influent and between 429 and 546 mg L^−1^ in the effluent, corresponding to a COD removal of ∼5.6%. These data indicate that heterotrophic denitrification took places in the SBR as commonly observed in unstable PN/A system, where biodegradable COD is available [22, 45]. A constant level of organic N (25 ± 2 mg N L^−1^) was detected in the effluent deriving from the amines constituting the dyes. Total P in the outlet was similar to the inlet 5.29 and 1.17, suspended solids decreased to 8–150 mg L^−1^ and conductivity was in the range of 1869–2870 μL.

### 3.2 Dynamic changes in microbial functional genes

**Figure 4:**
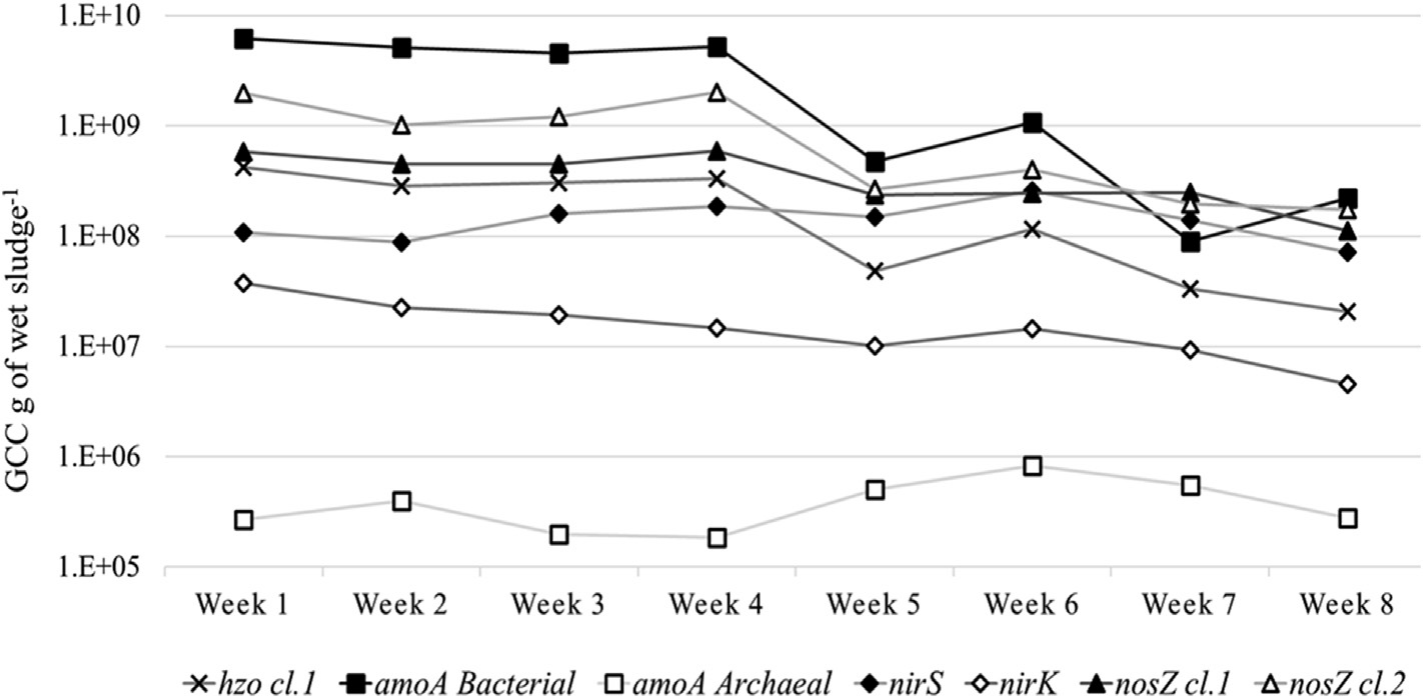
Variation in the gene copy concentrations (GCC g of wet sludge^−1^) across time of the nine analysed genes.

The abundance of the N related bacteria was assessed to evaluate the biochemical pathways occurring in the reactor. The patterns of gene copy concentration (GCCs) for each gene over time are shown in Figure 4.

A metanalysis study on PN/A bioreactors by [34] highlighted that 16S primers led to an underrepresentation of the total community due to the presence of very specific groups (e.g. anammox, comammox). Therefore, Orschler et al. [34] concluded that reporting qPCR data to the total 16S is not desirable in studies on PN/A bioreactors, and it is more appropriate to look at the dynamic variation for a gene. Within the same study, 16S primers specific for AMX showed that universal AMX primers are not suitable to analyse an unknown AMX community due to genus specificity within the AMX group [34, 46, 47].

Both the functional bacterial genes associated to AMX (*hzo*) and AOB (*amoA*) metabolisms ranged between 2.06×10^7^ to 4.19×10^8^ GCC per g of wet sludge and 8.99×10^7^ to 6.20×10^9^ GCC per g of wet sludge, respectively. Both gene copies concentrations significantly decreased of about 1 order of magnitude after the 5^th^ week of operation (*p value* < 0.001, one way ANOVA); *amoA* varied from 5.31×10^9^ ± 6.63×10^8^ GCC per g of wet sludge during week 1–4 to 4.64 ×10^8^ ± 4.34×10^8^ GCC per g of wet sludge in week 5–8, similarly *hzo* copy concentrations decreased from 3.35×10^8^ ± 5.92×10^7^ GCC per g of wet sludge to 5.46×10^7^ ± 4.24×10^7^ GCC per g of wet sludge. The decreasing trend of the AMX and AOB together with an increase of the NRR suggest that the PN/A process did not have a key role in the nitrogen removal process. Using the same primers amoA-1F/amoA-2R on two PN/A SBRs working at different operational conditions with domestic sewage, Wang et al. [48] found 1.21×10^7^ and 8.58×10^7^ GCC per g of dried sludge. While Gilbert et al. [49] reported values from 4.19×10^7^ to 3.97×10^8^ GCC ml^−1^, in a study on SBR reactors. In a PN/A reactor treating municipal landfill leachate, Sun et al. [50] found GCCs of 8.88×10^4^ - 1.82×10^8^ GCC ml^−1^ for *hzo* cluster1 (hzocl1F1/hzocl1R2).

A constant number of archaeal ammonia monooxygenase gene was also detected 4.03×10^5^ ± 2.2×10^5^ GCC per g of wet sludge, even though the significant low number of archaeal gene copies compared to the bacterial ones indicates their minor role in the ammonia oxidation process. AOA has been reported to co-exist and interact with AMX in several oxygen depleted natural and engineered environments (oxygen concentration about 5 μM) [51]. Overall, these findings suggest that the oxygen gradient within the granule allowed the establishment of ammonia oxidizing organisms with different niche requirements.

Denitrification related genes, *nirS* and *nirK*, averaged at 1.45×10^8^ and 1.67×10^7^ GCC per g of wet sludge, respectively; genes for the nitrous oxide reductase gene (*nosZ*) for clade 1 and 2 averaged 3.66×10^8^ and 9.07×10^8^ GCC per g of wet sludge, respectively. The suboptimal performance of the plant, which is demonstrated by the occurrence of denitrification and nitrite oxidation activity, reflected the community detected by molecular assays. Indeed, constantly high number of genes associated to denitrifiers were found with a slow decrease of the genes associated to AOB and anammox bacteria.

Pearson's correlation coefficients were used to assess the influence of the environmental parameters on the abundances of the genes (Table S4). Analysing the correlation between functional genes, it can be noticed that the AMX functional gene *hzo* showed a positive relationship with most denitrification (*nirK p value* < 0.05, *nosZ* cl1 and cl2 *p value* < 0.1) and bacterial nitrification genes (*amoA p value* < 0.001) while negative with archaeal (*amoA p value* < 0.05) suggesting a synergy among the three guilds of microbes. Additionally, both bacterial and archaeal *amoA* correlated, positively and negatively respectively, with *nosZ* clade 1 (*p value* < 0.01 and *p value* < 0.05 respectively) and clade 2 (both *p value* < 0.05). The AMX functional gene showed a positive relationship with the organic N in the influent (*p value* < 0.01). The organic N in the influent also correlated positively with *nosZ* (*p value* < 0.05 for both clades) and with the *amoA* gene for both bacteria (*p value* < 0.01) and archaea (*p value* < 0.05). The *amoA* gene correlated also negatively with the organic N in the effluent (*p value* < 0.05 for Archaea). Additionally, *nirS* correlated negatively (*p value* < 0.05) with COD in the effluent and pH in the influent while *nirK* correlated positively (*p value* < 0.05) with NH_4_^+^-N and the pH of the effluent. Denitrification, nitrification and anammox processes are highly dependent on temperature, pH, DO and C/N ratio and the effect of these parameters especially on AMX communities must be investigated individually to determine optimal conditions for each AMX system and pinpoint possible inhibition mechanisms [52, 53].

### 3.3 Dynamic changes in microbial diversity and composition

In order to understand how the community developed during the start-up period (first 4 weeks), the phylogenetic composition of the bacterial community was investigated using 16S rRNA gene high-throughput sequencing. Between 78,011 and 68,089 bacterial reads were initially retrieved after 16S rRNA analyses (40,431 - 33,241 after screening and bioinformatics) which resulted in a total of 343 amplicon sequence variants (ASVs) (198 ASVs for week 1, 224 for week 2, 196 for week 3, and 158 for week 4). These ASVs were divided in 16 phyla, 33 classes, 46 orders, 61 families, 74 genera, and 27 species.

**Figure 5:**
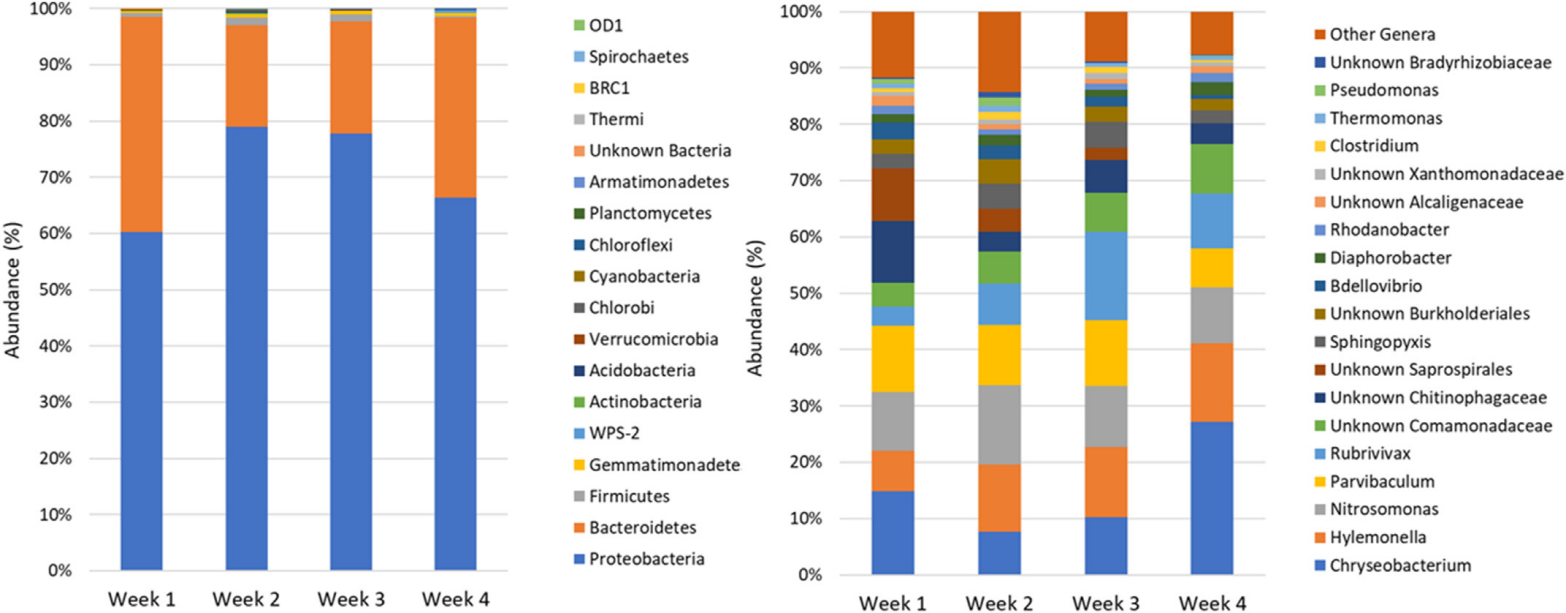
Left. Abundance of bacterial phyla for the four sampling times. Right. Abundance of bacterial genera present at abundances above 1% in at least one of the sludge samples for the four sampling times.

At a phylum level, *Proteobacteria* were the most dominant (60.3–79.0%) followed by *Bacteroidetes* (38.3–18.0%), *Firmicutes* (1.4–0.3%) and *Gemmatimonadetes* (0.7–0.2%) (Figure 5). *Actinobacteria* and *WPS-2* showed abundances of 0.3–0.1% and 0.6–0.1% respectively, while *Acidobacteria* was at 0.2–0.1%. Other phyla (*Verrucomicrobia*, *Chlorobi*, *Cyanobacteria*, *Chloroflexi*, *Planctomycetes*, *Armatimonadetes*, *Thermi*, *BRC2*, *Spirochaetes* and *OD2*) showed abundances below 0.1% within all dates and accounting together to 0.6–0.1% of the sequences. In general, on week 2 the system showed a higher diversity which decreased over time (Table S5).

Changes in the bacterial community structure at genera level were analysed for ASVs accounting for more than 1% of the relative abundance (Figure 5). NGS analysis further confirmed that the granules community was dominated by heterotrophic, nitrifying and denitrifying genera suggesting that shortcut nitrification-denitrification instead of PN/A process was established since the first weeks of operation. *Chryseobacterium* (genera containing species both capable of heterotrophic nitrification and aerobic denitrification [54, 55]) was the most common genera (7.7–27.2%), while other dominant genera were *Hylemonella* (7.3–13.9%, denitrifiers [56]), followed by *Nitrosomonas* (9.9–14.0%, mainly nitrifiers [57]), *Parvibaculum* (7.0–11.7%, aerobic hydrocarbon-degrading bacteria and denitrifiers [58, 59]), *Rubrivivax* (3.3–15.7%, involved in multiple biogeochemical and aromatic transformations, also denitrification, due to a wide metabolic capacity [58, 60]) and other *Comamonadaceae* (4.3–8.9%, aromatic degraders and denitrifiers [61]), *Chitinophagaceae* (3.6–10.9%), other *Saphrospirales* (0.1–9.4%), *Sphingopyxis* (2.2–4.7%), other *Burkholderiales* (2.1–4.3%), *Bdellovibrio* (0.7–2.9%), *Diaphorobacter* (1.2–2.2%, both heterotrophic nitrifiers and denitrifiers [62]), *Rhodanobacter* (1.0–1.6%), the families of *Alcaliganaceae* (0.7–1.7%) and *Xanthomonadaceae* (0.8–1.1%), *Clostridium* (0.3–1.3%, biopolymers degraders able of N fixation [63, 64]), *Thermomonas* (0.6–1.0%, denitrifiers [65, 66]), *Pseudomonas* (0.0–1.4%, capable of both heterotrophic nitrification and/or aerobic denitrification [67]), and the family of *Bradyrhizobiaceae* (0.2–1.0%, N fixation [68]), other bacteria that singularly accounted for less than 1% of the total abundance were 7.6–14.3%.

The group accounting for less than 1% of the relative abundance, included genera such as *Planctomyces* (0.0–0.1%) responsible for the anammox process [69], and bacteria belonging to the phyla of *Chloroflexi* (0.0–0.1%), *Chlorobi* (0.0–0.1%) and *Acidobacteria* (0.1–0.2%) known halotolerant and present in extreme and polluted environments [70]. Bacteria belonging to the genera of *Leucobacter* (0.0–0.3%) and *Hydrogenophaga* (0.2–0.5%) have been further found. These genera have been commonly found in textile wastewater due to their ability to degrade azo dyes [71, 72]. These two bacteria followed an opposite pattern, while *Leucobacter* abundance was highest during the second week and then decreased, *Hydrogenophaga* was lowest during the second week later showing a constant increase. Numerous studies further identified *Acinetobacter*, *Klebsiella* and *Pseudomonas* as three main genera including decolorizing bacteria and azo dyes degraders [24].

**Figure 6:**
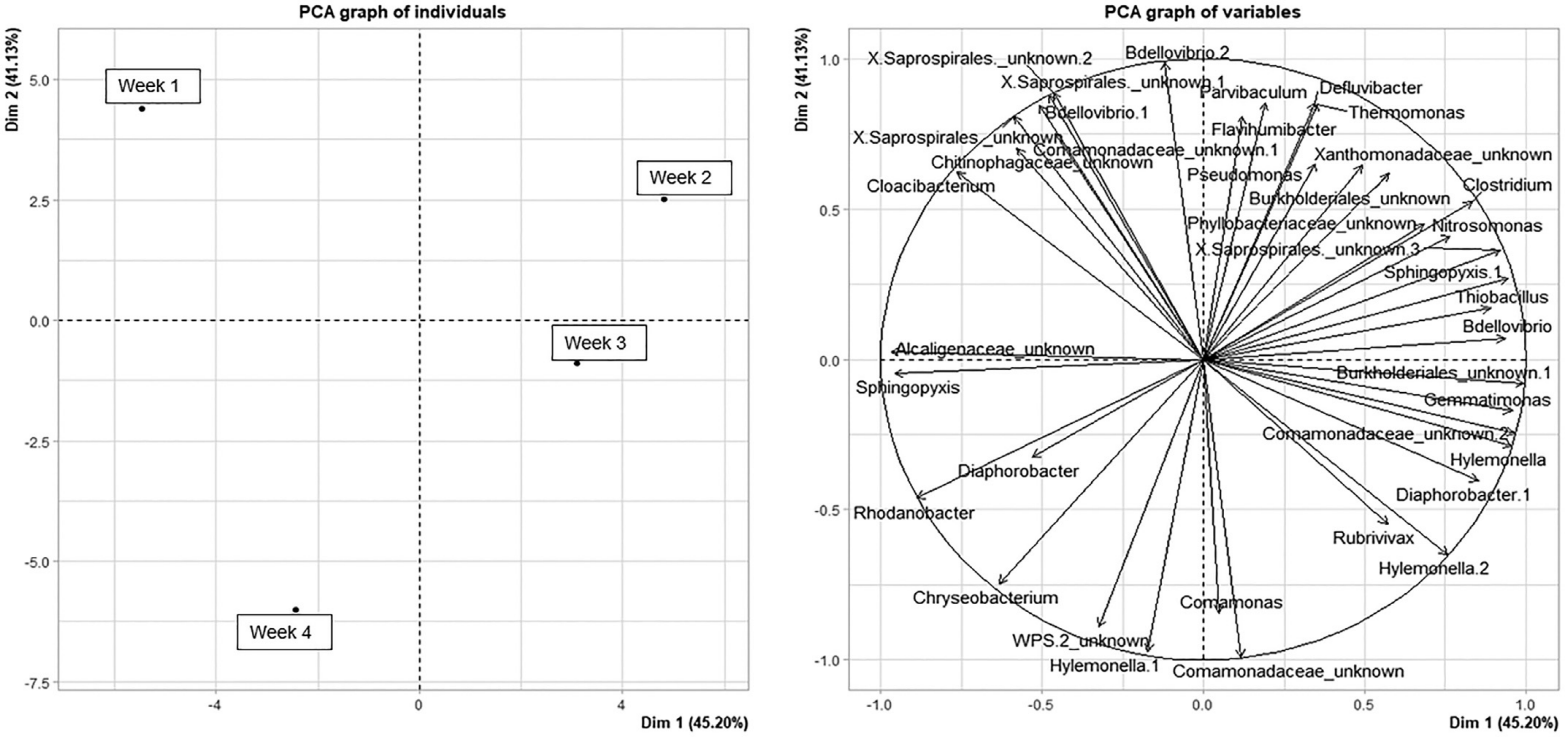
PCA of microbial community patterns of the four sampling times.

To find out the dynamic evolution of the bacterial community structure over time, a principal component analysis (PCA) was performed on genera that showed an abundance higher than 0.5% in at least one day (Figure 6). Across dimension 2, a temporal pattern was observed. Considering that length and orientation of the arrows is proportional to the direction and the amount of correlation between the ordination and the genera, at the beginning of the experiment (week 1), the community was prevalently characterised by the presence of several genera of *Saprospirales*, *Chitinophagaceae*, *Comamonadaceae*, *Bdellovibrio* and *Cloacibacterium*. The presence of *Bdellovibrio*, a bacterial predator, as influencing genera at this step could have significantly shaped the community possibly introducing a disturbance to the operational performance [73, 74]. On the next sampling date (week 2), the most significant genera were again belonging to the family of *Saprospirales* and *Phyllobacteriaceae*, *Nitrosomonas*, *Clostridium*, *Sphingopyxis* leading to an increase in AOB. On week 3, most defining genera were *Gemmatimonas*, a genus of *Burkholderiales* and *Comamonadaceae* while the week 4 was characterised by *Hylemonella*, *Chryseobacterium* and an unknown *WPS2* highlighting a possible higher role of denitrifiers during these two weeks.

### 3.4 Cost analyses and full-scale perspective

According to the annual average composition of the equalized wastewater (176 mg N/L, 784 mg COD/L, 225 mg BOD/L, 75 mg SST/L, 2.6 mg P/L), which was generated by the textile, the disposal cost to the centralized wastewater treatment plant is 2.25 €/m^3^ (855.686 €/y), while the cost of the treated wastewater (100 mg N/L, 493 mg COD/L, BOD 10 mg/L, 78.3 mg SST/L, 2.6 mg P/L) through the PN/Anammox based process, is 1.7 €/m^3^ (639 232 €/y); leading to a saving for the company of 0,57 €/m^3^, 216 454 €/y. These figures are calculated on the basis of the performance of the existing demonstrative SBR (treating capacity 40 m^3^/y), which was not optimal. Therefore, it is plausible that higher saving could be achieved by tailoring the operational conditions in order to decrease the N concentration in the effluents to 50 mg/L (∼1.3 €/m^3^, 40% of saving).

An economic assessment of the costs, capital and operational, required to scale up the SBR to a plant able to manage all the textile wastewater produced by the company (380 000 m^3^/y) was performed. The total investment for the construction of the plant is estimated to be 1 291 960 €. The major part of the investment is given by the construction work 1 152 000 €, while the design and construction management account for 81 880 € and 58 080 €, respectively. The start-up operational cost is 10 200 €, while the total annual operational cost amounts to 108 275 € y^−1^. Annual maintenance contributes for 38759 € y^−1^, consumption, including energy, for 28 120 € y^−1^, reagent for 13 737 € y^−1^, and labour for plant management accounts for 27 660 € y^−1^. The economic analyses were performed considering the geographical and market contexts of the textile company, which was examined in this study. It is representative of other Italian textile districts, while slight revisions should be made for areas, which differ for labour, energy and construction work prices.

## 4 Conclusions

The feasibility to treat real N-rich wastewater from textile digital printing through the PN/anammox process in a pilot scale SBR was evaluated. Results showed a satisfactory N removal efficiency (up to 70%), although the anammox activity was inhibited and denitrification occurred. The ratio between bacterial nitrification and anammox genes remained constant throughout the examined period, reflecting a constant ammonia oxidation activity by both organisms. Nevertheless, the metabarcoding analysis highlighted a prevalence of genera related to AOB and denitrifiers with a small portion of anammox bacteria suggesting that a shortcut nitrification-denitrification had a key role in the nitrogen removal process since the beginning of the trail.

Finally, this work revealed that PN/Anammox should be a promising cost-effective alternative for the treatment of N in textile wastewater allowing 50% saving in nitrogen removal costs compared with conventional treatment. The onsite implementation of a real scale SBR would permit the textile company to save up to 40% on wastewater disposal cost, though implying a conspicuous investment. However, the operational conditions and design of the proposed PN/Anammox based technologies should be better tailored to promote the growth and activity of anammox bacteria rather than the denitrifying bacteria. Continuous, instead of intermittent, operation of the reactor might mitigate the stress on the anammox bacteria caused by the toxicity and changes in the chemical and physical parameters of the textile wastewater. Also, shorter settling phase (shorter than 15 min) in the SBR could help reducing the abundance of the suspended growth biomass, which is mostly associated with heterotrophic denitrifiers.

## Declarations

### Author contribution statement

Elisa Clagnan and Micol Bellucci: Conceived and designed the experiments; Performed the experiments; Analyzed and interpreted the data; Wrote the paper.

Lorenzo Brusetti: Conceived and designed the experiments; Wrote the paper.

Silvia Pioli, Andrea Turolla, Mingsheng Jia, Elena Ficara, Roberto Canziani, and Giovanni Bergna: Analyzed and interpreted the data; Wrote the paper.

Simone Visigalli and Martina Bargna: Performed the experiments; Analyzed and interpreted the data; Wrote the paper.

### Funding statement

This work was supported by the EU’s LIFE Programme [project LIFE16ENV/IT/000345 “LIFE DeNTreat”], Fondazione Cariplo and Innovhub within the project “TRETILE” [project number 2017–1009]. This work was supported by the Open Access Publishing Fund of the Free University of Bozen-Bolzano.

### Data availability statement

All data generated or analysed during this study are included in this published article and its supplementary information files or are available in the NCBI SRA repository (BioProject accession number: PRJNA623880).

### Declaration of interests statement

The authors declare no conflict of interest.

### Additional information

No additional information is available for this paper.

## Appendix A Supplementary data

Supplementary Materials_ECF

## References

[1] Wang Z., Xue M., Huang KLiu Z., Hauser Peter (2011). Advances in Treating Textile Effluent.

[2] Kant R. (2012). Textile dyeing industry an environmental hazard. Nat. Sci..

[3] Emam H.E., Saad N.M., Abdallah A.E.M., Ahmed H.B. (2020). Acacia gum versus pectin in fabrication of catalytically active palladium nanoparticles for dye discoloration. Int. J. Biol. Macromol..

[4] Emam H.E., Ahmed H.B., Gomaa Helal M.H., Abdelhameed R.M. (2020). Recyclable photocatalyst composites based on Ag3VO4 and Ag2WO4 @MOF@cotton for effective discoloration of dye in visible light. Cellulose.

[5] Yang X., Zhou J., Huo T., Lv Y., Pan J., Chen L., Tang X., Zhao Y., Liu H., Gao Q., Liu S. (2021). Metabolic insights into the enhanced nitrogen removal of anammox by montmorillonite at reduced temperature. Chem. Eng. J..

[6] Medhi K., Gupta A., Takur S.I. (2019). Biological nitrogen removal from wastewater by Paracoccus denitrificans ISTOD1: optimization of process parameters using response surface methodology. J. Energy Environ. Sustain..

[7] Xia Y., Zhang M., Tsang D.C.W. (2020). Recent advances in control technologies for non-point source pollution with nitrogen and phosphorous from agricultural runoff: current practices and future prospects. Appl. Biol. Chem..

[8] Wang Y., Wang H., Wang X., Xiao Y., Zhou Y., Su X., Cai J., Sun F. (2020). Resuscitation, isolation and immobilization of bacterial species for efficient textile wastewater treatment: a critical review and update. Sci. Total Environ..

[9] Bellucci M., Marazzi F., Musatti A., Fornaroli R., Turolla A., Visigalli S., Bargna M., Bergna G., Canziani R., Mezzanotte Valeria., Rollini M., Ficara E. (2021). Assessment of anammox, microalgae and white-rot fungi-based processes for the treatment of textile wastewater. PLoS One.

[10] Beckinghausen A., Odlare M., Thorin E., Schwede S. (2020). From removal to recovery: an evaluation of nitrogen recovery techniques from wastewater. Appl. Energy.

[11] Scaglione D., Ficara E., Corbellini V., Tornotti G., Teli A., Canziani R., Malpei F. (2015). Autotrophic nitrogen removal by a two-step SBR process applied to mixed agro-digestate. Bioresour. Technol..

[12] Visigalli S., Turolla A., Bellandi G., Bellucci M., Clagnan E., Brusetti L., Jia M., Di Cosmo R., Menin G., Bargna M., Bergna G., Canziani R. (2020). Autotrophic nitrogen removal for decentralized treatment of ammonia-rich industrial textile wastewater: process assessment, stabilization and modelling. Environ. Sci. Pollut. Res..

[13] Li H., Wang S., Zhao F., Fang F., Chen Y., Yan P., Yang J., Gu S., Guo J. (2020). Evaluating the effects of micro-zones of granular sludge on one-stage partial nitritation–anammox nitrogen removal. Bioproc. Biosyst. Eng..

[14] Langone M., Yan J., Haaijer S.C.M., Op den Camp H.J.M., Jetten M.S.M., Andreottola G. (2014). Coexistence of nitrifying, anammox and denitrifying bacteria in a sequencing batch reactor. Front. Microbiol..

[15] Bagchi S., Lamendella R., Strutt S., Van Loosdrecht M.C.M., Saikaly P.E. (2016). Metatranscriptomics reveals the molecular mechanism of large granule formation in granular anammox reactor. Sci. Rep..

[16] Poot V., Hoekstra M., Geleijnse M.A.A., van Loosdrecht M.C.M., Pérez J. (2016). Effects of the residual ammonium concentration on NOB repression during partial nitritation with granular sludge. Water Res..

[17] Luo J., Chen H., Han X., Sun Y., Yuan Z., Guo J. (2017). Microbial community structure and biodiversity of size-fractionated granules in a partial nitritation – anammox process. FEMS Microbiol. Ecol..

[18] Hollocher T.C., Tate M.E., Nicholas D.J.D. (1981). Oxidation of ammonia by Nitrosomonas europaea: definitive ^18^O-tracer evidence that hydroxylamine formation involves a monooxygenase. J. Biol. Chem..

[19] Jubany I., Carrera J., Lafuente J., Baeza J.A. (2008). Start-up of a nitrification system with automatic control to treat highly concentrated ammonium wastewater: experimental results and modelling. Chem. Eng. J..

[20] Strous M., Kuenen J.G., Jetten M.S.M. (1999). Key physiology of anaerobic ammonium oxidation. Appl. Environ. Microbiol..

[21] Vlaeminck S.E., Cloetens L.F.F., Carballa M., Boon N., Verstraete W. (2008). Granular biomass capable of partial nitritation and anammox. Water Sci. Technol..

[22] Cho S., Kambey C., Nguyen V.K. (2020). Performance of anammox processes for wastewater treatment: a critical review on effects of operational conditions and environmental stresses. Water.

[23] Dosta J., Vila J., Sancho I., Basset N., Grifoll M., Mata-Álvarez J. (2015). Two-step partial nitritation/Anammox process in granulation reactors: start-up operation and microbial characterization. J. Environ. Manag..

[24] Meerbergen K., Van Geel M., Waud M., Willems K.A., Dewil R., Van Impe J., Appels L., Lievens B. (2017). Assessing the composition of microbial communities in textile wastewater treatment plants in comparison with municipal wastewater treatment plants. Open Microbiol. J..

[25] Yang Q., Wang J., Wang H., Chen X., Ren S., Li X., Xu Y., Zhang H., Li X. (2012). Evolution of the microbial community in a full-scale printing and dyeing wastewater treatment system. Bioresour. Technol..

[26] Yang Q., Wang J., Han X., Xu Y., Liu D., Hao H., Li X., Guo Y., Niu T., Qi S. (2014). Analysis of the bacterial community in a full-scale printing and dyeing wastewater treatment system based on T-RFLP and 454 pyrosequencing. Biotechnol. Bioproc. Eng..

[27] Thakur I.S., Medhi K. (2019). Nitrification and denitrification processes for mitigation of nitrous oxide from waste water treatment plants for biovalorization: challenges and opportunities. Bioresour. Technol..

[28] Wang Z.-B., Ni S.-Q., Zhang J., Zhu T., Ma Y.-G., Liu X.-L., Kong Q., Miao M.-S. (2016). Gene expression and biomarker discovery of anammox bacteria in different reactors. Biochem. Eng. J..

[29] Liu W., Marsh T.L., Cheng H., Forney L.J. (1997). Characterization of microbial diversity by determining terminal restriction fragment length polymorphisms of genes encoding 16S rRNA. Appl. Environ. Microbiol..

[30] Kim B.H., Gadd G.M. (2008).

[31] Philippot L., Bru D., Saby N.P.A., Čuhel J., Arrouays D., Šimek M., Hallin S. (2009). Spatial patterns of bacterial taxa in nature reflect ecological traits of deep branches of the 16S rRNA bacterial tree. Environ. Microbiol..

[32] Philippot L., Andert J., Jones C.M., Bru D., Hallin S. (2011). Importance of denitrifiers lacking the gene encoding the nitrous oxide reductase for N_2_O emissions from soil. Glob. Change Biol..

[33] Jones C.M., Graf D.R., Bru D., Philippot L., Hallin S. (2013). The unaccounted yet abundant nitrous oxidereducing microbial community: a potential nitrous oxide sink. ISME J..

[34] Orschler L., Agrawal S., Lackner S. (2019). On resolving ambiguities in microbial community analysis of partial nitritation anammox reactors. Sci. Rep..

[35] APHA, American Public Health Association (2005).

[36] Chelius M.K., Triplett E.W. (2001). The diversity of archaea and bacteria in association with the roots of Zea mays L. Microb. Ecol..

[37] Bonder M.J., Abeln S., Zaura E., Brandt B.W. (2012). Comparing clustering and pre-processing in taxonomy analysis. Bioinformatics.

[38] Callahan B.J., McMurdie P.J., Rosen M.J., Han A.W., Johnson A.J.A., Holmes S.P. (2016). DADA2: high-resolution sample inference from Illumina amplicon data. Nat. Methods.

[39] Jongman R.H.G., Ter Braak C.J.F., Van Tongeren O.F.R. (1995).

[40] Fukasawa Y., Osono T., Takeda H. (2009). Dynamics of physicochemical properties and occurrence of fungal fruit bodies during decomposition of coarse woody debris of Fagus crenata. J. For. Res..

[41] Autorità di Regolazione per Energia Reti e Ambiente (ARERA) - Deliberazione (28 Settembre 2017).

[42] de Almeida Fernandes L., Pereira A.D., Leal C.D., Davenport R., Werner D., Filho C.R.M., Bressani-Ribeiro T., de Lemos Chernicharo C.A., de Araújo J.C. (2018). Effect of temperature on microbial diversity and nitrogen removal performance of an anammox reactor treating anaerobically pretreated municipal wastewater. Bioresour. Technol..

[43] Duarte Pereira A., de Almeida Fernandes L., Campos Castro H.M., Dutra Leal C., Gonçalves Piteira Carvalho B., Dias M.F., Amaral Nascimento A.M., de Lemos Chernicharo C.A., Calábria de Araújo J. (2019). Nitrogen removal from food waste digestate using partial nitritation-anammox process: effect of different aeration strategies on performance and microbial community dynamics. J. Environ. Manag..

[44] Scaglione D., Lotti T., Menin G., Niccolini F., Malpei F., Canziani R. (2016). Complete autotrophic process for nitrogen removal from inkjet printing wastewater. Chem. Eng. Trans..

[45] Gonzalez-Martinez A., Muñoz-Palazon B., Rodriguez-Sanchez A., Gonzalez-Lopez J. (2018). New concepts in anammox processes for wastewater nitrogen removal: recent advances and future prospects. FEMS Microbiol. Lett..

[46] Zhou Z., Wei Q., Yang Y., Li M., Gu J.D. (2018). Practical applications of PCR primers in detection of anammox bacteria effectively from different types of samples. Appl. Microbiol. Biotechnol..

[47] Agrawal S., Weissbrodt D.G., Annavajhala M., Mark Jensen M., Carvajal Arroyo J.M., Wells G., Chandran K., Vlaeminck S.E., Terada A., Smets B.F., Lackner S. (2021). Time to act–assessing variations in qPCR analyses in biological nitrogen removal with examples from partial nitritation/anammox systems. Water Res..

[48] Wang Z., Zhang L., Zhang F., Jiang H., Ren S., Wang W., Peng Y. (2019). Nitrite accumulation in comammox-dominated nitrification-denitrification reactors: effects of DO concentration and hydroxylamine addition. J. Hazard Mater..

[49] Gilbert E.M., Agrawal S., Schwartz T., Horn H., Lackner S. (2015). Comparing different reactor configurations for Partial Nitritation/Anammox at low temperatures. Water Res..

[50] Sun F., Su X., Kang T., Wu S., Yuan M., Zhu J., Zhang X., Xu F., Wu W. (2016). Integrating landfill bioreactors, partial nitritation and anammox process for methane recovery and nitrogen removal from leachate. Sci. Rep..

[51] Straka L.L., Meinhardt K.A., Bollmann A., Stahl D.A., Winkler M.-K.H. (2019). Affinity informs environmental cooperation between ammonia-oxidizing archaea (AOA) and anaerobic ammonia-oxidizing (Anammox) bacteria. ISME J..

[52] Cho S., Kambey C., Nguyen V.K. (2020). Performance of anammox processes for wastewater treatment: a critical review on effects of operational conditions and environmental stresses. Water.

[53] You Q.-G., Wang J.-H., Qi G.-X., Zhou Y.-M., Guo Z.-W., Shen Y., Gao X. (2020). Anammox and partial denitrification coupling: a review. RSC Adv..

[54] Kundu P., Pramanik A., Dasgupta A., Mukherjee S., Mukherjee J. (2014). Simultaneous heterotrophic nitrification and aerobic denitrification by Chryseobacterium sp. R31 isolated from abattoir wastewater. BioMed Res. Int..

[55] Zhang Y., Fu C., Li X., Yan P., Shi T., Wu J., Wei X., Liu X. (2019). Optimizing suitable conditions for the removal of ammonium nitrogen by a microbe isolated from chicken manure. Open Chem..

[56] Tanikawa D., Yamashita S., Kataoka T., Sonaka H., Hirakata Y., Hatamoto M., Yamaguchi T. (2019). Non-aerated single-stage nitrogen removal using a down-flow hanging sponge reactor as post-treatment for nitrogen-rich wastewater treatment. Chemosphere.

[57] Koops H.-P., Pommerening-Röser A., Trujillo M.E., Dedysh S., DeVos P., Hedlund B., Kämpfer P., Rainey F.A., Whitman W.B. (2015). Bergey's Manual of Systematics of Archaea and Bacteria.

[58] Li E., Lu S. (2017). Denitrification processes and microbial communities in a sequencing batch reactor treating nanofiltration (NF) concentrate from coking wastewater. Water Sci. Technol..

[59] Ding J., Fu L., Lu Y., Ding Z., Zeng R.J. (2020). Evaluation of anaerobic ethane oxidation capability of the denitrifying anaerobic methane oxidation culture. Bioresour. Technol. Rep..

[60] Levy-Booth D.J., Hashimi A., Roccor R., Liu L.-Y., Renneckar S., Eltis L.D., Mohn W.W. (2021). Genomics and metatranscriptomics of biogeochemical cycling and degradation of lignin-derived aromatic compounds in thermal swamp sediment. ISME J..

[61] Khan S.T., Horiba Y., Yamamoto M., Hiraishi A. (2002). Members of the family Comamonadaceae as primary poly(3-hydroxybutyrate-co-3-hydroxyvalerate)-degrading denitrifiers in activated sludge as revealed by a polyphasic approach. Appl. Environ. Microbiol..

[62] Khardenavis A.A., Kapley A., Purohit H.J. (2007). Simultaneous nitrification and denitrification by diverse Diaphorobacter sp. Appl. Microbiol. Biotechnol..

[63] Chen J.-S., Klipp W., Masepohl B., Gallon J.R., Newton W.E. (2005). Dordrecht, Genetics and Regulation of Nitrogen Fixation in Free-Living Bacteria, Nitrogen Fixation: Origins, Applications, and Research Progress.

[64] Chu L., Wang J. (2016). Denitrification of groundwater using PHBV blends in packed bed reactors and the microbial diversity. Chemosphere.

[65] Chen H., Wang M., Chang S. (2020). Disentangling community structure of ecological system in activated sludge: core communities, functionality, and functional redundancy. Microb. Ecol..

[66] Ucar D., Yilmaz T., Di Capua F., Esposito G., Sahinkaya E. (2020). Comparison of biogenic and chemical sulfur as electron donors for autotrophic denitrification in sulfur-fed membrane bioreactor (SMBR). Bioresour. Technol..

[67] Lv P., Luo J., Zhuang X., Zhan D., Huang Z., Bai Z. (2017). Diversity of culturable aerobic denitrifying bacteria in the sediment, water and biofilms in Liangshui River of Beijing, China. Sci. Rep..

[68] Lindström K., Mousavi S.H. (2020). Effectiveness of nitrogen fixation in rhizobia. Microb. Biotechnol..

[69] Strous M., Pelletier E., Mangenot S., Rattei T., Lehner A., Taylor M.W., Horn M., Daims H., Bartol Mavel D., Wincker P., Barbe V.R., Fonknechten N., Vallenet D., Segurens B.A., Schenowitz-Truong C., Médigue C., Collingro A., Snel B., Dutilh B.E., Op den Camp H.J.M., van der Drift C., Cirpus I., van de Pas-Schoonen K.T., Harhangi H.R., van Niftrik L., Schmid M., Keltjens J., van de Vossenberg J., Kartal B., Meier H., Frishman D., Huynen M.A., Mewes H.W., Weissenbach J., Jetten M.S.M., Wagner M., Le Paslier D.A.T. (2006). Deciphering the evolution and metabolism of an anammox bacterium from a community genome. Nature.

[70] Canfora L., Bacci G., Pinzari F., Lo Papa G., Dazzi C., Benedetti A. (2014). Salinity and bacterial diversity: to what extent does the concentration of salt affect the bacterial community in a saline soil. PLoS One.

[71] Pandey A., Singh P., Iyengar L. (2007). Bacterial decolorization and degradation of azo dyes. Int. Biodeter. Biodegr..

[72] Franciscon E., Mendonça D., Seber S., Morales D.A., Zocolo G.J., Boldrin Zanoni M.V., Grossman M.J., Durrant L.R., Freeman H.S., Aragao Umbuzeiro G. (2015). Potential of a bacterial consortium to degrade azo dye Disperse Red 1 in a pilot scale anaerobic – aerobic reactor. Process Biochem..

[73] Feng S., Tan C.H., Constancias F., Kohli G.S., Cohen Y., Rice S.A. (2017). Predation by Bdellovibrio bacteriovorus significantly reduces viability and alters the microbial community composition of activated sludge flocs and granules. FEMS Microbiol. Ecol..

[74] Sun Y., Xin L., Wu G., Guan Y. (2019). Nitrogen removal, nitrous oxide emission and microbial community in sequencing batch and continuous-flow intermittent aeration processes. Environ. Eng. Res..

