# Supplementary material for: Microbial community and performance of a partial nitritation/anammox sequencing batch reactor treating textile wastewater
Source: Heliyon. 2021 Nov 20;7(11):e08445. doi: 10.1016/j.heliyon.2021.e08445 (PMC8637490; doi:10.1016/j.heliyon.2021.e08445)
Supplement: Supplementary file 1 — Supplementary Materials_ECF [file mmc1.docx]

**Supplementary materials**

**Fig. S1.** Total investment costs vs volumes of the treated wastewater (A) and the N load (B). The interpolations of the curves were used to compute the total investment costs needed for the construction of a PN/Anammox SBR plant in the textile company considered in the study.

**Table S1.** Genes and primer sets used for the qPCR of the sludge samples.

| **Genes**  **(target)** | **Primers** | **Amplicon size (bp)** | **qPCR condition** | **Reference** |
| --- | --- | --- | --- | --- |
| *amoA*  (AOB) | amoA-1F - amoA-2R | 491 | 95˚C-10 m; 45 cycles: 95˚C-1 m, 54˚C-1 m, acquisition at 72˚C-1 m; 72˚C-10 m; dissociation curve. [1] | [2] |
| *amoA*  (AOA) | amo19F - CrenamoA616r48x | 624 | 98 °C – 45 s; 55 °C – 45 s; 72 °C – 45 s; 40 cycles [3] | [4,5] |
| *hzo* cl1  (AMX) | hzoF - hzoR1 | 740 | 95˚C-10 m; 40 cycles: 95˚C-30 s, 56˚C-20 s, acquisition at 72˚C-40 s; 95˚C-15 s; dissociation curve [6] | [6] |
| *nirK*  *(Denitrifying bacteria)* | nirK876 - nirK1040 | 164 | 95˚C-15 min; 6 cycles: 95˚C-15 s, 63 to 58˚C-30 s with a decrease of 1˚C every cycle, 72˚C-30 s, 80˚C-15 s; 40 cycles:95˚C-15 s, 60˚C-30 s, 72˚C-30 s, acquisition at 80˚C-30 sec; 95˚C-15 sec; dissociation curve [7] | [8] |
| *nirS*  *(Denitrifying bacteria)* | Cd3aF - R3cd | 416 | 95˚C-10 m; 40 cycles: 95˚C-30 s, 57˚C-20 s, acquisition at 72˚C-30 s; 95˚C-15 s; dissociation curve [9] | [10,11] |
| *nosZ* cl1  *(Denitrifying bacteria)* | nosZ2F - nosZ2R | 267 | 95˚C-15 m; 6 cycles: 95˚C-15 s, 65 to 60˚C-30 s with a decrease of 1˚C every cycle, 72˚C-30 s, 80˚C-15 s; 40 cycles: 95˚C-15 s, 60˚C-15 s, 72˚C-30 s, acquisition at 80˚C-30 s; 95˚C-15 s; dissociation curve [12] | [12] |
| *nosZ* cl2  *(Denitrifying bacteria)* | nosZ-II-F - nosZ-II-R | 683 | 95˚C-15 m; 40 cycles: 95˚C-15 s, 60˚C-30 s, 72˚C-30 s acquisition at 80˚C-30 s; 95˚C-15 s; dissociation curve. [13] | [13] |

**Table S2.** Costs for the construction of an SBR with the PN/Anammox process in function of the scale and design. In the context of CAPEX, the investment required as a function of the volume of the wastewater or N load was inferred: the costs of civil and electromechanical work, of six potential plants differing in dimensions were estimated on the basis of the design (reactor volume, treatment lines, N load, ect.).

| Volume (m^3^/y) | N load (Kg/d) | Reactor Volume (m^3^) | Reactor Line | Volume/Line  (m^3^) | Civil work (1000 €) | Electro-mechanical work  (1000 €) | Total  (1000 €) |
| --- | --- | --- | --- | --- | --- | --- | --- |
| 400000 | 303 | 500 | 4 | 125 | 300 | 1000 | **1300** |
| 200000 | 152 | 250 | 2 | 125 | 210 | 770 | **980** |
| 100000 | 76 | 125 | 1 | 125 | 150 | 590 | **740** |
| 50000 | 38 | 72 | 4 | 15 | 50 | 500 | **550** |
| 25000 | 19 | 36 | 2 | 15 | 35 | 350 | **385** |
| 12500 | 10 | 18 | 1 | 15 | 25 | 250 | **275** |

**Table S3.** Costs required to operate the full scale SBR.

| **People** |  |
| --- | --- |
| *Costs* |  |
| Technical people (€/h) | 50 |
| Operating personnel (€/h) | 35 |
| *Working hour for plant start up* |  |
| Technical people (h/plant) | 120 |
| Operating personnel (h/plant) | 120 |
| ***Working hour for plant management*** |  |
| Technical people (h/month/plant) | 16 |
| Operating personnel (h/month/plant) | 43 |
| **Overheads** |  |
| On people, material and services (%) | 2 |
| **Annual maintenance** |  |
| Material on Investment (%) | 2 |
| Services on investment (%) | 1 |
| **Annual Consumption** |  |
| Material on investment (%) | 1 |
| Electric energy* (€/m3) | 0.04 |
| **Reagents** |  |
| Anti-foam (€/m3) | 0.006 |
| Acid / Base (€/m3) | 0.03 |
| Water (€/m3) | 0.00015 |

* Assuming 0.25 kWh/m3 and the specific cost of 0.16 €/kWh.

**Table S4.** Correlations among environmental variables and GCCs (* p<0.05, **p<0.01, ***p<0.001).

|  | **hzo cl.1** |  | **amoA**  **Bacterial** | **amoA**  **Archaeal** | **nirS** | **nirK** | **nosZ cl.1** | **nosZ cl.2** | **NH_4_^+^-N_IN_** | **COD_IN_** | **Organic N_IN_** | **NO_2_^-^-N_OUT_** | **NO_3_^-^-N_OUT_** | **NH_4_^+^-N_OUT_** | **COD**  **_OUT_** | **Organic N**  **_OUT_** | **pH**  **_IN_** | **pH**  **_OUT_** | **Concucibility**  **_IN_** | **Concucibility**  **_OUT_** | **Suspended solids**  **_IN_** | **Suspended solids**  **_OUT_** | **Total P**  **_IN_** |  | **Total P**  **_OUT_** |
| --- | --- | --- | --- | --- | --- | --- | --- | --- | --- | --- | --- | --- | --- | --- | --- | --- | --- | --- | --- | --- | --- | --- | --- | --- | --- |
| **hzo cl.1** |  |  |  |  |  |  |  |  |  |  |  |  |  |  |  |  |  |  |  |  |  |  |  |  |  |
| **amoA Bacterial** | 0.99*** |  |  |  |  |  |  |  |  |  |  |  |  |  |  |  |  |  |  |  |  |  |  |  |  |
| **amoA Archaeal** | -0.87* |  | -0.84* |  |  |  |  |  |  |  |  |  |  |  |  |  |  |  |  |  |  |  |  |  |  |
| **nirS** | -0.15 |  | -0.21 | -0.32 |  |  |  |  |  |  |  |  |  |  |  |  |  |  |  |  |  |  |  |  |  |
| **nirK** | 0.82* |  | 0.79 | -0.48 | -0.57 |  |  |  |  |  |  |  |  |  |  |  |  |  |  |  |  |  |  |  |  |
| **nosZ cl.1** | 0.96** |  | 0.95** | -0.89* | 0.02 | 0.69 |  |  |  |  |  |  |  |  |  |  |  |  |  |  |  |  |  |  |  |
| **nosZ cl.2** | 0.94** |  | 0.91* | -0.89* | 0.1 | 0.68 | 0.99*** |  |  |  |  |  |  |  |  |  |  |  |  |  |  |  |  |  |  |
| **NH_4_^+^-N_IN_** | -0.72 |  | -0.74 | 0.55 | 0.45 | -0.74 | -0.53 | -0.5 |  |  |  |  |  |  |  |  |  |  |  |  |  |  |  |  |  |
| **COD_IN_** | -0.49 |  | -0.58 | 0.51 | 0.15 | -0.24 | -0.39 | -0.32 | 0.79 |  |  |  |  |  |  |  |  |  |  |  |  |  |  |  |  |
| **Organic N_IN_** | 0.92** |  | 0.96** | -0.85* | -0.07 | 0.64 | 0.91* | 0.88* | -0.75 | -0.72 |  |  |  |  |  |  |  |  |  |  |  |  |  |  |  |
| **NO_2_^-^-N_OUT_** | 0.38 |  | 0.48 | -0.12 | -0.71 | 0.42 | 0.2 | 0.08 | -0.7 | -0.73 | 0.45 |  |  |  |  |  |  |  |  |  |  |  |  |  |  |
| **NO_3_^-^-N_OUT_** | 0.35 |  | 0.38 | -0.54 | 0.1 | 0.07 | 0.23 | 0.16 | -0.51 | -0.69 | 0.39 | 0.55 |  |  |  |  |  |  |  |  |  |  |  |  |  |
| **NH_4_^+^-N_OUT_** | 0.69 |  | 0.63 | -0.43 | -0.4 | 0.91* | 0.58 | 0.62 | -0.7 | -0.17 | 0.55 | 0.16 | -0.11 |  |  |  |  |  |  |  |  |  |  |  |  |
| **COD_OUT_** | 0.32 |  | 0.29 | 0.13 | -0.83* | 0.8 | 0.17 | 0.16 | -0.43 | 0.13 | 0.11 | 0.33 | -0.31 | 0.74 |  |  |  |  |  |  |  |  |  |  |  |
| **Organic N _OUT_** | 0.57 |  | 0.49 | -0.85* | 0.64 | 0.23 | 0.62 | 0.7 | -0.32 | -0.27 | 0.57 | -0.32 | 0.32 | 0.39 | -0.29 |  |  |  |  |  |  |  |  |  |  |
| **pH_IN_** | 0.54 |  | 0.6 | -0.08 | -0.84* | 0.76 | 0.46 | 0.39 | -0.47 | -0.18 | 0.45 | 0.64 | -0.11 | 0.52 | 0.78 | -0.35 |  |  |  |  |  |  |  |  |  |
| **pH_OUT_** | 0.74 |  | 0.77 | -0.35 | -0.68 | 0.89* | 0.66 | 0.62 | -0.71 | -0.37 | 0.7 | 0.54 | -0.09 | 0.8 | 0.75 | 0.03 | 0.88* |  |  |  |  |  |  |  |  |
| **Concucibility _IN_** | -0.42 |  | -0.42 | -0.04 | 0.78 | -0.75 | -0.35 | -0.31 | 0.24 | -0.19 | -0.2 | -0.36 | 0.3 | -0.54 | -0.87* | 0.36 | -0.92** | -0.75 |  |  |  |  |  |  |  |
| **Concucibility_OUT_** | 0.13 |  | 0.11 | -0.02 | -0.35 | 0.38 | -0.08 | -0.05 | -0.71 | -0.47 | 0.19 | 0.34 | 0.16 | 0.58 | 0.39 | 0.15 | 0.05 | 0.35 | 0.06 |  |  |  |  |  |  |
| **Suspended solids _IN_** | 0.5 |  | 0.52 | -0.7 | 0.57 | -0.01 | 0.68 | 0.7 | -0.06 | -0.32 | 0.66 | -0.2 | 0.07 | 0.03 | -0.49 | 0.65 | -0.11 | 0.12 | 0.27 | -0.31 |  |  |  |  |  |
| **Suspended_solids_OUT_** | 0.59 |  | 0.63 | -0.3 | -0.57 | 0.73 | 0.46 | 0.45 | -0.85* | -0.61 | 0.67 | 0.56 | 0.02 | 0.78 | 0.58 | 0.14 | 0.6 | 0.87* | -0.39 | 0.72 | 0.1 |  |  |  |  |
| **Total P_IN_** | -0.28 |  | -0.33 | 0.2 | 0.47 | -0.33 | -0.05 | 0.03 | 0.78 | 0.76 | -0.33 | -0.85* | -0.76 | -0.2 | -0.2 | 0.05 | -0.25 | -0.28 | 0.03 | -0.62 | 0.33 | -0.47 |  |  |  |
| **Total P_OUT_** | -0.79 |  | -0.83* | 0.84* | -0.08 | -0.38 | -0.8 | -0.72 | 0.48 | 0.6 | -0.8 | -0.47 | -0.68 | -0.12 | 0.16 | -0.45 | -0.31 | -0.34 | 0.11 | 0.23 | -0.57 | -0.18 | 0.35 |  |  |

**Table S5.** Richness and diversity indexes calculated on the whole dataset.

|  | **Richness** | **Shannon** | **Shannon.effective** | **Simpson** | **Simpson.effective** | **Evenness** |
| --- | --- | --- | --- | --- | --- | --- |
| **Week 1** | 198 | 3.50 | 33.19 | 0.07 | 15.24 | 0.46 |
| **Week 2** | 224 | 3.74 | 42.15 | 0.05 | 19.49 | 0.48 |
| **Week 3** | 196 | 3.32 | 27.60 | 0.07 | 13.84 | 0.44 |
| **Week 4** | 158 | 2.98 | 19.60 | 0.11 | 8.94 | 0.41 |

# Supplementary References

[1] Segal LM, Miller DN, McGhee RP, Loecke TD, Cook KL, Shapiro CA, Drijber RA. Bacterial and archaeal ammonia oxidizers respond differently to long-term tillage and fertilizer management at a continuous maize site. Soil Till. Res. 2017;168:110-117 <https://doi.org/10.1016/j.still.2016.12.014>

[2] Rotthauwe J, Witzel K, Liesack W. The ammonia monooxygenase structural gene amoA as a functional marker: Molecular fine-scale analysis of natural ammonia-oxidizing populations. Appl. Environ. Microbiol. 1997;63:4704-4712. <https://doi.org/10.1128/AEM.63.12.4704-4712.1997>

[3] Harter J, Krause HM, Schuettler S, Ruser R, Fromme M, Scholten T, Kappler A, Behrens S. Linking N_2_O emissions from biochar-amended soil to the structure and function of the N-cycling microbial community. ISME J. 2014;8:660–674. <https://doi.org/10.1038/ismej.2013.160>

[4] Leininger S, Urich T, Schloter M, Schwark L, Qi J, Nicol GW, Prosser JI, Schuster SC, Schleper C. Archaea predominate among ammonia-oxidizing prokaryotes in soils. Nature 2006;442:806–809. <https://doi.org/10.1038/nature04983>

[5] Schauss K, Focks A, Leininger S, Kotzerke A, Heuer H, Thiele-Bruhn S, Sharma S, Wilke BM, Matthies M, Smalla K, Munch JC, Amelung W, Kaupenjohann M, Schloter M, Schleper C. Dynamics and functional relevance of ammonia-oxidizing archaea in two agricultural soils. Environ Microbiol. 2009;11:446–456. <https://doi.org/10.1111/j.1462-2920.2008.01783.x>

[6] Kong L, Jing H, Kataoka T, Buchwald C, Liu H. Diversity and Spatial Distribution of Hydrazine Oxidoreductase (hzo) Gene in the Oxygen Minimum Zone Off Costa Rica. PLOS One, 2013;8(10):e78275. <https://doi.org/10.1371/journal.pone.0078275>

[7] Henry S, Baudoin E, Lopez-Gutierrez JC, Martin-Laurent F, Brauman A, Philippot L. Quantification of denitrifying bacteria in soils by nirK gene targeted real-time PCR. J. Microbiol. Methods. 2004;59:327-335. Corrigendum 2005;61:289-290.

[8] Hallin S, Jones CM, Schloter M, Philippot L. Relationship between N-cycling communities and ecosystem functioning in a 50-year-old fertilization experiment. ISME J. 2009;3(5):597-605. <https://doi.org/10.1038/ismej.2008.128>

[9] Thompson KA, Bent E, Abalos D, Wagner-Riddle C, Dunfield KE. Soil microbial communities as potential regulators of in situ N_2_O fluxes in annual and perennial cropping systems. Soil Biol. Biochem. 2016;103: 262-273. <https://doi.org/10.1016/j.soilbio.2016.08.030>

[10] Michotey V, Mejean V, Bonin P. Comparison of methods for quantification of cytochrome cd(1)-denitrifying bacteria in environmental marine samples. Appl. Environ. Microbiol. 2000;66:1564- 1571. <https://doi.org/10.1128/aem.66.4.1564-1571.2000>

[11] Throback IN, Enwall K, Jarvis A, Hallin S. Re-assessing PCR primers targeting nirS, nirK and nosZ genes for community surveys of denitrifying bacteria with DGGE. FEMS Microbiol. Ecol. 2004;49:401-417. <https://doi.org/10.1016/j.femsec.2004.04.011>

[12] Henry S, Bru D, Stres B, Hallet S, Philippot L. Quantitative detection of the nosZ gene, encoding nitrous oxide reductase, and comparison of the abundances of 16S rRNA, narG, nirK, and nosZ genes in soils. Appl. Environ. Microbiol. 2006;72 (8):5181-5189. <https://doi.org/10.1128/AEM.00231-06>

[13] Jones CM, Graf DR, Bru D, Philippot L, Hallin S. The unaccounted yet abundant nitrous oxidereducing microbial community: a potential nitrous oxide sink. ISME J. 2013;7(2):417–426. <https://doi.org/10.1038/ismej.2012.125>
